# Potential Promises and Perils of Human Biological Treatments for Immunotherapy in Veterinary Oncology

**DOI:** 10.3390/vetsci10050336

**Published:** 2023-05-09

**Authors:** Jeilene N. Hambly, Carl E. Ruby, Dan V. Mourich, Shay Bracha, Brian P. Dolan

**Affiliations:** 1 Department of Biomedical Sciences, Carlson College of Veterinary Medicine, Oregon State University, Corvallis, OR 97331, USA; 2 Biotesserae Inc., Corvallis, OR 97331, USA; 3 Department of Veterinary Clinical Sciences, College of Veterinary Medicine, The Ohio State University, Columbus, OH 43210, USA

**Keywords:** tumor immunotherapy, single-domain antibody, comparative oncology, checkpoint inhibitor, tumor vaccines

## Abstract

**Simple Summary:**

Immunotherapy for the treatment of cancer seeks to use the body’s own immune system to recognize and eliminate tumor cells. Some of the most successful immunotherapies in human medicine have relied on the generation of biological reagents specific to humans, such as tumor antigen vaccines and humanized monoclonal antibodies. Veterinarians would like to incorporate immunotherapies into oncogenic medicine; however, the readily available reagents are biological therapies designed for human use, and their utility in veterinary medicine remains unknown. In some instances, such as tumor antigen vaccination, humanized reagents may prove advantageous in animal species. In other instances, such as the use of humanized monoclonal antibodies, the treatment may fail as a result of the animal’s own immune system rejecting the human reagent. Here, we review the potential use of these reagents for veterinary oncology and explore other possible reagents that may have “universal” applicability in different animal species.

**Abstract:**

The emergence of immunotherapy for the treatment of human cancers has heralded a new era in oncology, one that is making its way into the veterinary clinic. As the immune system of many animal species commonly seen by veterinarians is similar to humans, there is great hope for the translation of human therapies into veterinary oncology. The simplest approach for veterinarians would be to adopt existing reagents that have been developed for human medicine, due to the potential of reduced cost and the time it takes to develop a new drug. However, this strategy may not always prove to be effective and safe with regard to certain drug platforms. Here, we review current therapeutic strategies that could exploit human reagents in veterinary medicine and also those therapies which may prove detrimental when human-specific biological molecules are used in veterinary oncology. In keeping with a One Health framework, we also discuss the potential use of single-domain antibodies (sdAbs) derived from camelid species (also known as Nanobodies™) for therapies targeting multiple veterinary animal patients without the need for species-specific reformulation. Such reagents would not only benefit the health of our veterinary species but could also guide human medicine by studying the effects of outbred animals that develop spontaneous tumors, a more relevant model of human diseases compared to traditional laboratory rodent models.

## 1. Introduction

Veterinary medicine often adopts therapeutic strategies developed for human medicine to treat similar diseases in animal patients. These treatments often include medications approved for use in humans that target similar physiological pathways in animals. For example, the most common agents used for the treatment of veterinary oncological diseases are cytotoxic chemotherapies, such as alkylating agents (e.g., cyclophosphamaide), vinca alkaloids (e.g., Vincristine), anthracyclines (e.g., Doxorubicin) and platinum-based drugs (e.g., Carboplatin), which were originally developed for human cancer patients. The strategy has shown benefit to the veterinary profession, as bypassing novel drug development saves both time and funding. While this strategy may prove successful when using small (or even large) pharmaceutical chemicals, it is more problematic when considering the use of biological agents, which are often tailored specifically for the use in a single species. Nowhere is this more important than in the recently emerging field of tumor immunotherapy, which relies on activating or reinvigorating the patient’s own immune system to recognize and remove transformed cells while sparing healthy tissue. Mostly developed for use in human medicine, the principles involved in immunotherapy are expected to translate into veterinary medicine [1]. However, the currently existing reagents themselves may or may not yield the desired results. It, therefore, behooves us to consider when and where human biological reagents can be adapted directly to the veterinary clinic and when they should be avoided.

## 2. Tumor Immunotherapy: General Principles

The goal of using the body’s own immune system to destroy transformed cells is over a century old; however, successfully harnessing the immune system to fight cancer has remained elusive [2]. In their seminal 1957 paper, Prehn and Main began by stating “The history of attempts to immunize against cancer is one of long frustration [3]”, a phrase which, until the last decade or so, could have been the introductory sentence to numerous PhD theses on tumor immunology. Since then, immunotherapy has moved from a once unattainable goal to the first-line treatment option for a growing number of specific cancers [4].

What has changed in recent years to spur on this paradigm shift in cancer treatment? There have been several important discoveries made, which demonstrate why immunotherapy can be effective. Tumors are derived from “self” tissues; thus, the prior prevailing thought in the field was that tumor reactive T-cells would be deleted by the positive thymic selection of lymphocytes in order to reduce the risk of autoimmune reactions. However, the thinking in the field shifted, as it became known that tumor cells can harbor specific antigens that the immune system can target. There are several classes of these tumor-associated antigens [5], including overexpressed self-proteins [6,7] and unique new antigens, known as neoantigens, created as a result of continuous mutations, which occur during gene reorganization and tumor progression [8,9]. Neoantigens, in particular, are attractive targets for the immune system, as T-cells can be capable of recognizing such antigens that make the tumor appear foreign or non-self. The presence of neoantigens and other associated tumor antigens has spurred on the development of tumor antigen vaccines, which could be used to therapeutically treat oncologic diseases [10,11]. Therefore, even though they are derived from self-tissues, tumor cells can, in fact, harbor the antigens that mark them as targets for the adaptive immune response.

Going hand-in-hand with the continuous discovery of tumor antigens is the increased appreciation for cytotoxic T-cells in eliminating cancer cells [12,13]. Cytotoxic T-cells are responsible for eliminating self-cells that have become infected or transformed. To accomplish this, T-cells must be able to recognize that a cell in the body is diseased, a process which involves the presentation of disease-derived peptides on major histocompatibility complex (MHC) class I molecules. In addition to detecting a disease-associated peptide, cytotoxic T-cells also receive secondary signals from target cells and the target cell’s environment, which can further activate or, alternatively, inhibit T-cell-mediated killing [14]. Negative signals, such as checkpoint signals, delivered to T-cells are necessary in order to control the immune response and prevent an unnecessary or potentially dangerous autoimmune response; however, tumors often hijack the expression of checkpoint molecules to thwart cytotoxic T-cell responses [15,16]. Blocking these checkpoint signaling pathways is, therefore, an attractive way to overcome a T-cell-suppressive environment in the tumor and reestablish tumor-specific cytotoxic T-cell responses against cancer cells.

These findings provided the drive to push immunologists and clinicians to develop and test new ways to boost anti-tumor immune responses in the clinic, with impressive results. Numerous strategies have been adopted to prime and enhance the adaptive immune system’s ability to recognize and kill tumor cells, such as vaccination with tumor-specific antigens [17], in vitro expansion and reintroduction of immune cells capable of supporting anti-tumor responses [18], development of monoclonal antibodies, which can directly target tumor cells for destruction [19], genetically engineering autologous T-cells to react with tumor-specific proteins on the cell surface [20] and the development of monoclonal antibody therapies, which can block negative signals delivered from the tumor microenvironment to T-cells [21]. As a result, immunotherapy is emerging to be the first-line option for oncologists [4].

Veterinary oncologists, looking at the success of human immunotherapy, will adopt some of these strategies for animal patients. Some treatments, such as the expansion of autologous patient immune cells or the generation of chimeric-antigen receptor (CAR) T-cells, will likely be too expensive for clinical applications, at least in the near future. Treatment options that are not cell-based therapies offer a more cost-effective and simpler option; however, many of the reagents used in immunotherapy are biological molecules and are developed specifically for use in humans. Considerations of how biologic-based reagents will work in a different species will be needed before this existing suite of therapeutic products developed for humans can be used safely and effectively in the veterinary clinic. Below, we will review some of the existing products in human medicine and describe which therapies may have advantages in veterinary species and which may not.

## 3. Human Tumor Antigen Vaccination

One branch of immunotherapy incorporates our understanding of tumor-associated antigens to develop vaccines, which target said antigens. Tumor antigens could be introduced to the patient using multiple strategies, such as vaccination with purified proteins or peptides mixed with adjuvants, using recombinant attenuated pathogens to deliver antigens or by the introduction of nucleic acid sequences encoding the tumor antigen [22]. The recent successes obtained through the use of mRNA vaccines to drive protective immune responses against SARS-CoV-2 across the globe have demonstrated the utility in the use of nucleic-acid-based vaccines to successfully prime the immune system to recognize the introduced antigen. It is, therefore, plausible that we will see more and more nucleic acid vaccines targeting tumor antigens in the future.

The orthologs of several human tumor-associated antigens have also been identified in companion animals, and these proteins are often present in similar tumor types as in humans. Many of these potential targets can be found in melanomas, which is important as canine melanomas are a model for human melanomas [23]. Bergman and colleagues set out to determine if tyrosinase could be an immune target in canine melanoma [24]. To test this, dogs were vaccinated with a readily available human DNA plasmid vaccine that encodes human tyrosinase (hTyr). The amino acid similarity between hTyr and canine Tyr is 91%, which suggests that the differences in amino acid sequences could overcome any adaptive immune tolerance [24]. Initial clinical studies demonstrated potential benefits, including extended survival times and the induction of anti-tyrosinase antibodies, which suggest that a xeongenic tumor-associated antigen could serve as a target to generate clinically effective anti-tumor T-cell responses [24,25,26].

Several clinical trials and retrospective studies were subsequently conducted with the DNA-based hTyr vaccine, sold under the name Oncept^®^, for the treatment of melanoma [27,28,29,30,31,32]. Some studies showed a relative degree of efficacy [27,28], though it should be noted that others did not show a benefit [29]. Most of these studies were retrospective studies and may not accurately capture the true impact of a drug treatment [27,28,29]. Nevertheless, Oncept is safe and well-tolerated, and as future studies are conducted, it is likely that the effect of prognostic indicators, such as tumor size and lymph node metastasis, on Oncept efficacy will be better detailed [32]. A recent case report also reported that Oncept can be combined with a tyrosine kinase inhibitor to treat lingual metastatic melanoma [33], raising the exciting possibility of a combinational approach with a vaccine and other adjuvant therapies, including chemotherapy, to elicit better clinical outcomes. Therefore, xenogenic vaccination with DNA-encoded plasmids is a potential approach for using existing therapies developed for humans in a veterinary oncology setting.

Other melanoma-associated tumor antigens could also be the target of xenogenic vaccination. Chondroitin sulfate proteoglycan-4 (CSPG-4) is another potential antigenic target in multiple tumor types [34]. Human CSPG-4 is very similar to canine CSPG-4 (82% homology and 88% similarity) and would, therefore, make an excellent choice of antigens [35]. A DNA-based vaccine encoding hCSPG-4 has been used in multiple clinic trials to induce adaptive immune responses against cCSPG-4 in dogs with melanoma, and the initial results appear promising [35,36,37]. Riccardo et al. tested hCSPG-4 vaccination in dogs with stage II-III oral malignant melanoma and noted extended 6- and 12-month survival times following surgical resection and vaccination compared to animals who received surgery alone, accompanied by an increase in anti-CSPG-4 antibodies in serum [35]. The authors used a technique referred to as electrovaccination, where, immediately following the injection of plasmid DNA into the muscle, a series of short electrical pulses were applied to the injection site to boost plasmid DNA uptake into the nucleus of cells [38]. A second follow-up study, by Piras et al., confirmed the extended survival times and reduced lung metastasis in vaccinated dogs compared to unvaccinated controls [36]. In addition, a retrospective study also suggested that vaccination with hCSPG-4 can increase survival time, especially when initial surgery focused on curative intent rather than cytoreduction only [37]. Taken together, these studies suggest that xenogeneic vaccination with hCSPG-4 in canine patients following surgical removal of melanoma tumors can enhance survival time by possibly driving an anti-tumor CSPG-4-specific response [35,36,37].

Xenogeneic DNA vaccines are not the only method to introduce human antigenic proteins into veterinary patients. Recombinant pathogens, such as viruses and bacteria, can be engineered to express antigenic proteins when infecting a host, often driving impressive immune responses [22,39]. Recent studies have demonstrated the potential efficacy of using recombinant Listeria monocytogenes bacteria to deliver tumor antigens to hosts [40]. Due to the nature of the bacteria, L. monocytogenes infections result in the secretion of bacterial antigens into the host cell cytoplasm, which are subsequently presented by MHC class I molecules in a highly efficient manner [41,42]. There are currently several human clinical studies investigating the use of Lm-vaccines to induce anti-tumor immune responses [40].

In veterinary species, an Lm-based vaccine for the treatment of osteosarcoma (OSA) is currently in clinical trials [43]. Osteosarcoma is the most common bone neoplasia in dogs, accounting for 85% of bone tumors [44], and is thought to have immunogenic tumor properties [45,46,47]. When local disease is controlled, pulmonary metastasis is thought to be the biggest contributor to life-limiting disease processes [48,49]. Canine osteosarcoma has many similarities with human osteosarcoma [50,51,52,53,54], as it is the most common bone tumor and commonly affects larger breeds and taller individuals [47,55]. Additionally, the proto-oncogene erbB-2, also known as Her-2, has been found to be overexpressed in canine osteosarcoma, similar to human osteosarcoma [56]. Thus, canine osteosarcoma disease is of interest not only for therapeutic treatments in companion animals but also for translation purposes in human oncology. Therapeutic improvements in both the human and veterinary clinics for long-term treatment options are needed, as the current standard of care has not changed in over 20 years. Immunotherapy options are promising but have yet to be fully understood [47].

ADXS31-164 is an Lm-based vaccine vector expressing human Her-2 that was developed originally for use in human patients [57] but is now also being considered for use in canine patients with osteosarcoma [43]. In a clinical trial, enrolling 18 dogs that had a primary tumor removed via amputation and received chemotherapy, ADXS31-164 administration resulted in increased median survival time compared to historical controls [43]. Several of the dogs in the clinical trial also showed evidence of increased Her-2-specific T-cell responses following vaccination [43]. The vaccine is available in lyophilized form, and a recent study noted some adverse events in dogs receiving the vaccine [58], although it should be noted that some animals tested positive for Listeria following vaccination [58].

**Figure 1:**
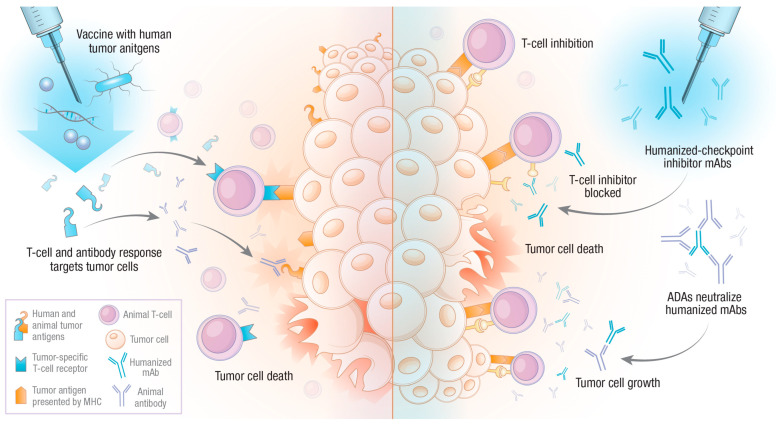
Potential outcomes of humanized biological reagents in veterinary oncology. The administration of humanized biological compounds to veterinary patients will lead to an adaptive immune response against xenogenic reagents. In the case of tumor antigen vaccination (**left side**), the result may be deemed successful if the animal’s adaptive immune response can cross-react with the orthologous antigen or break tolerance. However, animal adaptive immune forces may also recognize humanized monoclonal antibodies, for instance, checkpoint inhibitors (**right side**). This can lead to anti-drug antibody (ADA) responses, which neutralize therapeutic antibodies, resulting in a T-cell inhibitory state, allowing for tumor growth.

Combined, the data from these studies suggest a potential benefit for the use of a humanized agent in veterinary medicine. By using a human tumor-associated antigen, an adaptive immune response is generated that recognizes the orthologous animal antigen, perhaps even breaking previously existing immune tolerance to the targeted protein. It is likely that the slight differences between the human and animal proteins are enough to initiate an adaptive immune response, which, in the case of vaccination, is of benefit to the patient. However, as we will see in the next section, those same differences can lead to undesired immune responses to other therapies (see Figure 1).

## 4. Humanized Monoclonal Antibodies

Some of the most exciting results of human immunotherapies come from the use of checkpoint inhibitors. These reagents are monoclonal antibodies, which specifically block the interaction of suppressive signaling molecules on T-cells with their cognate ligands, ensuring that cytotoxic T-cells can be maintained in an active state and allowing for the killing of tumor cells [59]. To date, the most effective checkpoint inhibitor drug targets are CTLA-4, the PD-1/PD-L1 signaling pathways and LAG-3, which can be targeted by a recent FDA-approved drug, Relatlimab [60]. Relatlimab joins eight other monoclonal antibodies approved by the FDA to treat a variety of cancer types. The impact of these therapies on the field of oncology cannot be understated and is the subject of many excellent reviews [21,61,62].

Orthologs of negative stimulators of T-cells are annotated in the genomes of many vertebrate species available in the NCBI database. Additionally, the expression of these proteins has been detected in the tumors of companion animals. For instance, PD-1/PD-L1 expression has been shown to be upregulated in a number of different canine tumors [63,64,65,66]. Monoclonal antibodies, which detect canine PD-1/PD-L1, have been developed [65,67,68,69], which could be useful for both diagnostic and therapeutic use in the veterinary clinic. Furthermore, blocking the PD-1 and PD-L1 axis in vitro with mAbs antibodies can enhance T-cell function [63,65,67], as can blocking CTLA-4 [70]. Additionally, perhaps most excitingly, several FDA-approved checkpoint inhibitor antibodies can recognize and even block canine PD-1/PD-L1 interactions in vitro with atezolizumab (anti-PD-L1), showing the most robust increase in the production of the activation marker IFNγ [71]. Therefore, at least in dogs, and likely in cats [72], negative T-cell signaling pathways exist, are present in tumors and can be targeted with monoclonal antibodies to enhance T-cell function. However, can the checkpoint inhibitor antibodies developed for human use be administered in other animals?

Antibodies have been used clinically for over 100 years, even before their identity was known and their functions were completely understood. Often, antibodies for use in human medicine were generated in other mammalian species. For instance, patients with diptheria were often treated with diptheria antitoxin (DAT), a polyclonal antibody solution derived from horses inoculated with Cornybacterium diphteriae, which neutralizes the diptheria toxin produced during infection [73]. However, the administration of DAT requires careful monitoring to be sure that patients do not develop hypersensitivity reactions [73]. The reader is encouraged to review Silverstein’s paper detailing Pirquet’s description of serum sickness from the early 1900s [74], where Pirquet correctly identified the cause of serum sickness occurring when the patient recognizes the transferred antibody as a foreign agent, and the body mounts its own adaptive immune response to target the therapeutic antibody. The development of antibodies that recognize these exogenous therapeutic immunoglobulins are referred to as anti-drug antibodies (ADAs).

While the first descriptions of ADAs were in response to adoptively transferred polyclonal antibodies in sera, ADAs can also be detected in highly purified preparations of monoclonal antibodies. The first monoclonal antibody therapy approved for use in humans in the United States was muromonab CD3, a mouse monoclonal antibody targeting CD3 and widely used to suppress immune responses in transplant patients [75]. However, recipients often developed ADAs, which induced side effects [76,77]. Future generations of monoclonal antibody therapies partially solved this problem using the introduction of chimeric monoclonal antibodies: recombinant antibodies where most of the constant region of the mouse monoclonal antibody is replaced with a human Fc. However, even these chimeric antibodies still elicited ADAs. For instance, infliximab, a chimeric mAb that targets TNFα to prevent inflammatory reactions, is often immunogenic in patients. In fact, ADA responses can require an increasing dose of infliximab during treatment to maintain its efficacy [78,79,80,81]. In some instances, chimeric mAb can induce hypersensitivity responses. Cetuximab is a chimeric mAb that binds to the epithelial growth factor receptor (EGFR) on cancer cells to target the cells for destruction [82]. However, cetuximab treatment can result in hypersensitivity reactions in patients with an atopic history [83]. This has been linked to patient IgE antibodies, which can target a particular glycan, galactose-alpha-1,3-galactose (alpha-gal) [84], a common antigenic target in patients who exhibit Alpha-gal Syndrome (AGS), where an intolerance to mammalian-derived food products develops [85].

ADA responses generated following the adoptive transfer of fully murine or even chimeric antibodies can lower the efficacy of the drug, or worse, create or exacerbate hypersensitivity reactions [86] in humans. Therefore, it is likely that the use of human monoclonal antibodies in veterinary patients will almost certainly induce similar ADA responses. ADA responses have been documented in dogs treated with monoclonal 231 (mAb231), a murine-based antibody therapy developed in the 1980s for treating lymphomas in dogs [87,88]. In human medicine, it has become increasingly apparent that there is a need to fully “humanize” monoclonal antibodies to reduce the likelihood of generating ADAs following treatment [89]. Many of the current immunotherapies in use consist of humanized antibodies, where only the amino acid sequence of the antigen-specific CDRs remain in the final product.

To circumvent the anti-drug antibody responses elicited in veterinary patients, antibody-based checkpoint inhibitor therapies will need to be re-engineered for each species of interest. The caninization of antibodies, where the constant domain of the (usually) rodent monoclonal antibody is replaced with the constant region of canine antibodies, has been reported. For instance, the anti-IL-31 antibody Lokivetmab, which is used in the treatment of atopic and allergic dermatitis [90,91,92], is a fully caninized monoclonal antibody. Anti-nerve growth factor mAbs have also been adapted for both canine and feline patients [93,94,95]. Fully caninized antibodies that could be used as checkpoint inhibitors are currently being developed. Recent pilot studies were conducted with caninized anti-PD-1 and anti-PD-L1 antibodies, which showed the caninized antibodies to be relatively safe [96,97]. Participant numbers were limited, but preliminary evidence also suggested a potential benefit to treatment with canonized anti PD-1 antibodies [96]. Studies with anti-PD-L1 antibodies suggest limited clinical benefit [98,99]. A caninized anti-CTLA-4 has also been developed and has been shown to enhance in vitro T-cell function [70]. While it is likely that caninized and felinized monoclonal antibodies will be developed in the future, a great deal of work will need to be undertaken to understand the functions and distributions of FcRs in each veterinary species of interest in order to successfully incorporate monoclonal antibody therapies. The speciation of checkpoint inhibitor antibodies will be of little use if these therapeutics trigger massive histamine release or activate the complement cascade upon binding to their target, which may occur if the wrong Fc portion of an antibody is coupled to a PD-1 or CTLA-4 targeting CDR.

Because of the potential for poorer target recognition, the triggering of anti-drug antibody responses and the lack of detailed knowledge regarding veterinary FcR usage and distribution, the development of checkpoint inhibitor therapy for veterinary species remains a daunting task. While future research should certainly attempt to tackle this problem, other treatment options may offer the advantage of increased safety to use in veterinary species.

## 5. Single-Domain Antibodies (sdAbs)

**Figure 2:**
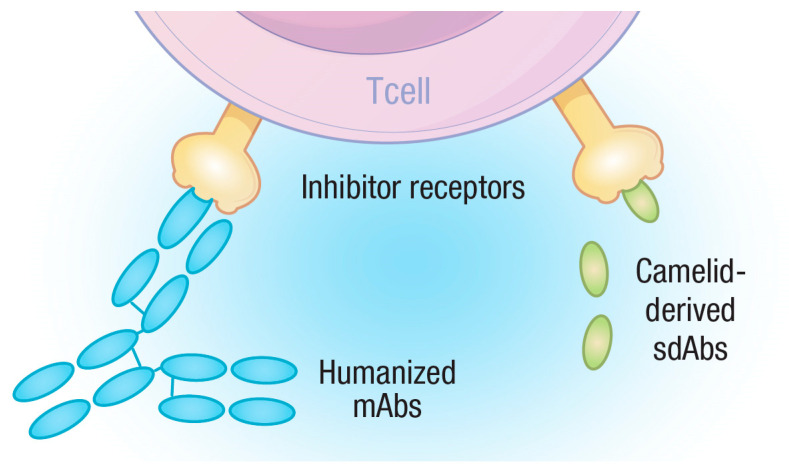
Diagram depicting the rationale for sdAbs as checkpoint inhibitors. Monoclonal antibodies consist of two heterodimers containing multiple domains while sdAbs have a single domain. Both mAbs and sdAbs can recognize and bind to targets, such as T-cell inhibitory receptors, blocking their ability to negatively regulate T-cells, but sdAbs present far fewer epitopes for the adaptive immune system to target and are, therefore, less likely to be subjected to ADA responses.

Studying the immune system in different animal species can often yield surprising and useful findings, perhaps none more so than the discovery of single-chain antibodies, first identified in camelid species [100] and later in cartilaginous fish [101], in an interesting case of divergent evolution. Camelid single-chain antibodies have captured the imagination of the biotech world, as they offer up a much simpler way to produce proteins with antibody-like specificity but through the production of a single protein, instead of both a heavy- and light-chain immunoglobulin [102,103]. Furthermore, a single immunoglobulin domain containing the CDR can be produced, which has the ability to recognize its target antigen but carries none of the remaining Fc region of the immunoglobulin. These molecules are known as single-domain antibodies (sdAbs) and often referred to as Nanobodies™. These reagents offer the ability to target a molecule with antibody-like specificity but without the potential downstream hazards of antibody therapies that can be related to FcR interactions. From a veterinarians’ perspective, single-domain antibodies hold great promise for the simple reason that they could be used in multiple species without the need for species-specific reformulation, as would be necessary for the more complex monoclonal antibodies. We will, therefore, describe, in more detail, the emerging literature regarding the use of sdAbs in the clinic, paying particular attention to their use in checkpoint inhibitor therapy (Figure 2).

### 5.1. The Advantage of Being Small

Immunoglobulin G (IgG) molecules, which make up nearly all classes of therapeutic monoclonal antibodies, are roughly ~140 kDa in size and consist of four proteins, two heavy-chain and two light-chain proteins, tethered together by several disulfide bonds. While IgG molecules are present in sera and can migrate into tissues, their relatively large size places limits on their utility. For instance, it has been known for decades that monoclonal antibodies poorly infuse solid tumors [104,105,106,107]. In contrast, sdAbs, which consist of a single immunoglobulin domain, are significantly smaller, with an average molecular weight of 15–16 kDa [108]. This size differential provides sdAbs with advantages over classical antibodies. The compact size of an sdAb allows it to access more compact bodily spaces, such as organs and solid tumors. When compared directly to conventional antibodies, sdAbs tend to accumulate in tumors with faster kinetics [109,110,111]. Thus, the use of sdAbs will aid several hard-to-treat diseases compared to the more traditional antibody.

In addition to penetrating diverse tissues, the small size of sdAbs also allows for quicker clearance from the body following injection [109]. This reduces the chances of off-target effects of the sdAbs, which will be clinically important if an sdAb is being used to deliver radioactive compounds to visualize tumors or toxic payloads to kill cancer cells. Furthermore, the dose of sdAbs required to achieve a therapeutic outcome is less than more traditional antibodies. SdAbs may also enhance the tumor penetration of classic antibodies [112].

Finally, the small size of an sdAb limits the number of antigenic targets that could be introduced through the introduction of a xenogenic protein. By eliminating nearly 85% of the amino acid sequence of a monoclonal antibody, none of which is necessary for interacting with the antigenic target of the antibody, we can eliminate most immune-induced anti-drug responses. Indeed, studies in humans suggest a limited immunogenicity of sdAbs, though it should be noted that the literature is rather sparse [113,114]. SdAbs in clinical trials can be taken up by dendritic cells but cause minimal activation [114]. Rossotti et al. expertly reviewed the recent clinical and pre-clinical reports, which collectively suggest that even when ADAs develop following sdAb infusion, there appears to be a limited loss of efficacy [113]. However, there are two notable exceptions [115,116], which suggest that the ADAs to sdAbs may preclude their use in the clinic. In a surprising and non-intuitive manner, the sdAbs in question were more “humanized” versions than other camelid-derived sdAbs used in the clinic [115,116]. This observation is vexing, and clearly future work is needed to understand the ADAs to sdAbs. Veterinary medicine could help fill this gap in knowledge by testing sdAbs in a variety of species to determine the extent of ADA responses to sdAbs.

### 5.2. Towards Clinical Applications

The time for sdAb use in many different clinical applications is fast approaching. Already, several clinical trials using sdAbs for the treatment of a variety of diseases are underway. For a comprehensive list of the most updated reviews on the subject, the reader is encouraged to see [117,118,119]. The first clinical trials to utilize sdAbs targeted the Von Willebrand factor, marketed as caplacizumab. Caplacizumab is now routinely used to treat thrombotic thrombocytopenic purpura [120,121]. Several other sdAbs targeting immune molecules, such as TNFα, IL-17 and the IL6-R, are in clinical or preclinical development for the treatment of a diversity of diseases [122,123,124].

The adaptation of sdAb technology as a checkpoint inhibitor has obvious importance in not only animal medicine but human medicine as well. Targeting CTLA-4 or PD-1 in solid tumors has remained challenging, but the use of an sdAb in place of an antibody may allow for greater drug permeability into solid tumors, promoting the cytotoxic T-cell killing of tumor cells. Several recent studies have identified potential sdAbs, which may act as checkpoint inhibitors, primarily the PD-1/PD-L1 axis [125,126,127]. Thus, it is entirely plausible that checkpoint inhibitor sdAbs could supplement or even replace existing antibody-based therapies in the future [128,129,130].

Perhaps most importantly, checkpoint inhibitor sdAbs might be readily transitioned into the veterinary clinic with limited modifications for the treatment of tumors in a variety of species. Not only would this greatly expand the arsenal of veterinary oncologists to treat patients but it could serve as a unique opportunity for comparative oncology studies. In addition to the previously discussed oral malignant melanoma and osteosarcoma, several cancers in companion animals are close mimics of human disease, such as canine multicentric lymphoma [131,132,133], canine invasive urothelial cell carcinoma [134,135,136,137] and feline mammary carcinoma [72,138,139]. In some instances, they are considered the best animal models for particular cancers. One could imagine clinical trials that combine checkpoint inhibitor therapy with more traditional chemotherapies in spontaneously occurring tumors in naturally outbred species, which closely mimic human disease. Not only would such trials benefit companion animal health but they could also provide very valuable information to inform human medicine, and they would occur at a fraction of the cost of human clinical trials. It is, therefore, imperative that the field not only develops checkpoint inhibitor sdAbs but also quickly ascertains their potential for use in veterinary species. Initial clinical trials in veterinary patients should not only determine any potential toxicity related to the administration of sdAbs but also determine if patients generate an ADA directed against asAbs.

## 6. In the Future

The promise of immunotherapy for the treatment of veterinary tumors is on the horizon; however, significant hurdles remain. Chief among them is the expense and time taken for drug development, which can often limit treatment options in animal species. Using off-the-shelf products intended for human use can eliminate some of these hurdles. In some instances, such as vaccination with human tumor antigens, the xenogenic differences will prove to be beneficial, as the slight differences between amino acid sequences in the target protein can successfully drive adaptive immune responses. However, this can be a double-edged sword as the same adaptive immune forces can target humanized reagents, such as checkpoint inhibitor antibodies, and, thus, induce immune-mediated anti-drug responses, which will, at best, eliminate drug efficacy and, at worst, lead to immune-induced hypersensitivity reactions. While it is possible to create species-specific checkpoint inhibitor monoclonal antibodies, a more exciting and useful approach may lie in the development of checkpoint inhibitor sdAbs, which are far more likely to be utilized in multiple species. The field needs to urgently explore this potentially groundbreaking therapy, which in a true One Health framework and will not only benefit animal health but can also teach us about human health.

## Data Availability

No new data were created or analyzed in this study. Data sharing is not applicable to this article.
